# Epoetin alpha prevents anaemia and reduces transfusion requirements in patients undergoing primarily platinum-based chemotherapy for small cell lung cancer

**DOI:** 10.1038/sj.bjc.6690369

**Published:** 1999-05-01

**Authors:** N Thatcher, E S De Campos, D R Bell, W P Steward, G Varghese, R Morant, J F Vansteenkiste, R Rosso, S-B Ewers, E Sundal, E Schatzmann, H Stocker

**Affiliations:** CRC Department of Oncology, Christie Hospital NHS Trust, Wilmslow Road, Manchester M20 4BX, UK; Royal North Hospital, Sydney, Australia; Leicester Royal Infirmary, Leicester, UK; Belfast City Hospital, Belfast, UK; Kantonsspital, St Gallen, Switzerland; University Hospital Gasthuisberg, Leuven, Belgium; Medical Oncology Institute, Genova, Italy; University Hospital, Lund, Sweden; Janssen-Cilag, Oslo, Norway; RW Johnson Pharmaceutical Research Institute, Bassersdorf, Switzerland

**Keywords:** epoetin prevents chemotherapy SCLC anaemia

## Abstract

Anaemia commonly occurs in cancer patients receiving chemotherapy, often necessitating blood transfusion. This multicentre study was designed to evaluate the efficacy and safety of epoetin α in preventing the decline in haemoglobin (Hb) level, and to determine whether the transfusion requirement could be reduced, in patients receiving 4–6 cycles of primarily platinum-based combination cyclic chemotherapy for small cell lung cancer (SCLC). A total of 130 non-anaemic SCLC patients were randomized to receive no additional treatment (*n* = 44), epoetin α 150 IU kg^−1^ subcutaneously (s.c.) three times a week (*n* = 42) or 300 IU kg^−1^ s.c. three times a week (*n* = 44). Reductions in epoetin α dosage were made during the study if Hb level increased to >15 g dl^−1^. The mean weekly dosage was 335 and 612 IU kg^−1^, respectively, in the two active treatment groups. Significantly fewer (*P* < 0.05) epoetin α-treated patients experienced anaemia (Hb < 10 g dl^−1^) during the course of chemotherapy (300 IU kg^−1^, 39%; 150 IU kg^−1^, 48%; untreated, 66%). This was reflected in the significantly lower number of treated patients transfused [300 IU kg^−1^, 20% (*P* < 0.001); 150 IU kg^−1^, 45% (*P* < 0.05); untreated, 59%]. Epoetin α was well-tolerated, and there was no evidence of sustained, clinically significant, hypertension. In summary, epoetin α is effective and well-tolerated in maintaining Hb level and reducing transfusion requirement in patients undergoing cyclic chemotherapy for SCLC. © 1999 Cancer Research Campaign


*British Journal of Cancer* (1999) **80**(3/4), 396–402
© 1999 Cancer Research Campaign
Article no. bjoc.1998.0369

### Epoetin alpha prevents anaemia and reduces transfusion requirements in patients undergoing primarily platinum-based chemotherapy for small cell lung cancer

**N Thatcher[1], ES De Campos[1], DR Bell[2], WP Steward[3], G Varghese[4], R Morant[5], JF Vansteenkiste[6], R Rosso[7], S-B Ewers[8], E Sundal[9], E Schatzmann[10] and H Stocker[10]**

[1]CRC Department of Oncology, Christie Hospital NHS Trust, Wilmslow Road, Manchester M20 4BX, UK; [2]Royal North Hospital, Sydney, Australia; [3]Leicester Royal Infirmary, Leicester, UK; [4]Belfast City Hospital, Belfast, UK; [5]Kantonsspital, St Gallen, Switzerland; [6]University Hospital Gasthuisberg, Leuven, Belgium; [7]Medical Oncology Institute, Genova, Italy; [8]University Hospital, Lund, Sweden; [9]Janssen-Cilag, Oslo, Norway; [10]RW Johnson Pharmaceutical Research Institute, Bassersdorf, Switzerland

**Summary** Anaemia commonly occurs in cancer patients receiving chemotherapy, often necessitating blood transfusion. This multicentre study was designed to evaluate the efficacy and safety of epoetin α in preventing the decline in haemoglobin (Hb) level, and to determine whether the transfusion requirement could be reduced, in patients receiving 4–6 cycles of primarily platinum-based combination cyclic chemotherapy for small cell lung cancer (SCLC). A total of 130 non-anaemic SCLC patients were randomized to receive no additional treatment ($n$ = 44), epoetin α 150 IU kg$^{-1}$ subcutaneously (s.c.) three times a week ($n$ = 42) or 300 IU kg$^{-1}$ s.c. three times a week ($n$ = 44). Reductions in epoetin α dosage were made during the study if Hb level increased to >15 g dl$^{-1}$. The mean weekly dosage was 335 and 612 IU kg$^{-1}$, respectively, in the two active treatment groups. Significantly fewer ($P$ < 0.05) epoetin α-treated patients experienced anaemia (Hb < 10 g dl$^{-1}$) during the course of chemotherapy (300 IU kg$^{-1}$, 39%; 150 IU kg$^{-1}$, 48%; untreated, 66%). This was reflected in the significantly lower number of treated patients transfused [300 IU kg$^{-1}$, 20% ($P$ < 0.001); 150 IU kg$^{-1}$, 45% ($P$ < 0.05); untreated, 59%]. Epoetin α was well-tolerated, and there was no evidence of sustained, clinically significant, hypertension. In summary, epoetin α is effective and well-tolerated in maintaining Hb level and reducing transfusion requirement in patients undergoing cyclic chemotherapy for SCLC.

**Keywords:** epoetin prevents chemotherapy SCLC anaemia

Cytotoxic chemotherapy is one of the principal causes of anaemia in patients with cancer. It may become progressively worse with repeated cycles of chemotherapy (Kuzur and Greco, 1980), and frequently aggravates pre-existing anaemia that is a common occurrence in these patients. Thus, patients may experience an overall worsening of their condition as a result of the additional debilitating symptoms of anaemia. Palliative transfusions are often administered in an attempt to ameliorate some of these effects. However, allogeneic transfusion per se is associated with a risk of adverse effects, such as bacterial, viral contamination, iron overload, rash, itching and other reactions (Walker, 1987). There is also some evidence that blood transfusion can suppress the immune system, which in turn influences the immune surveillance of cancer and cancer recurrence rates (Blumberg and Heal, 1994).

Chemotherapy regimens containing cisplatin or carboplatin are particularly associated with a high requirement for transfusions. Moderate-to-severe anaemia occurs in 10–40% of patients treated with cisplatin (Hesketh et al, 1988; Gebbia et al, 1990), although the incidence may be increased still further with high dosages. In a recent study by Kaye et al (1992), for example, transfusions were required by 53% of patients treated with a median total cisplatin dose of 500 mg m$^{-2}$ for ovarian cancer. Moreover, anaemia appears to occur in a similar percentage of patients treated with carboplatin (59%) (Canetta et al, 1985).

Endogenous erythropoietin (EPO) production in response to anaemia is known to be inadequate in anaemic cancer patients compared with patients with iron deficiency anaemia. This relative lack of EPO is further exacerbated by cytotoxic chemotherapy (Miller et al, 1990). Similarly, blunted EPO responses are seen in patients with non-cancer-related anaemia of chronic disease (ACD), such as rheumatoid arthritis (Baer et al, 1987) but not in those with anaemia due to other causes such as iron deficiency or acute blood loss. It has been speculated that the maturation and proliferation of EPO-producing cells may be directly affected by the effects of nephrotoxic chemotherapeutic agents such as cisplatin, thereby reducing EPO production (Rothman et al, 1985; Miller et al, 1990). However, anaemia (in some cases severe) has been reported in cisplatin-treated patients with no evidence of underlying renal failure (Rothman et al, 1985). Also, no difference has been observed in the EPO response to anaemia in patients treated with cisplatin- and non-cisplatin-containing regimens. Mechanisms other than nephrotoxicity may therefore cause the blunting of the EPO response (Rothmann et al, 1985).

Several studies in anaemic chemotherapy-treated patients have shown increased haemoglobin (Hb) levels and decreased red blood cell (RBC) transfusion requirements following treatment with

*Received 26 March 1998*
*Revised 24 June 1998*
*Accepted 27 June 1998*

*Correspondence to:* N Thatcher epoetin α in patients with a variety of malignant diseases; moreover, epoetin α was well-tolerated (Henry et al, 1989; Ludwig et al, 1990, 1993*a*, 1993*b*; Abels, 1993; Cascinu et al, 1993; Case et al, 1993). More recently a non-randomized phase IV study with data available on 2030 patients was reported. Epoetin α resulted in a 50% decrease in the number of patients requiring transfusion independent of malignancy type and a reduction in the number of units of red cells transfused per patient per month (Glaspy et al, 1997). Prophylactic epoetin α studies have had relatively little attention in advanced solid malignancies. A small randomized study of 62 patients with early stage breast cancer receiving chemotherapy demonstrated that Hb levels were maintained close to baseline levels in epoetin-treated patients whereas in the control group there was progressive anaemia (Del Mastro et al, 1997).

Recent studies have indicated that lung cancer is associated with a particularly high requirement for transfusion following chemotherapy (Skillings et al, 1993), possibly because of poor tolerance of anaemia in this patient population. A subgroup of the total study population presented in the Results section was analysed to determine the effects of epoetin α on erythroid progenitors in the bone marrow. Essentially, the findings were that the erythroid progenitors were reduced in the marrow of small cell lung cancer (SCLC) patients compared with non-malignant controls. Epoetin increased the level of erythroid progenitors when assayed 1 month after chemotherapy had been completed compared with a non-epoetin α-treated patient group (de Campos et al, 1995). The aim of the current study was to determine the efficacy and safety of epoetin α in preventing the decline in Hb level in patients undergoing cyclic chemotherapy for SCLC, and to evaluate whether a reduction in RBC transfusion requirements could also be achieved. The impact of epoetin α therapy on patients' quality of life (QOL) was also assessed.

### PATIENTS AND METHODS

Male or female patients (aged 18–75 years), planned for treatment with 4–6 cycles of combination chemotherapy, primarily platinum-based, for SCLC were enrolled in this open-label, randomized, parallel-group, multicentre study. Chosen chemotherapy regimens and doses were at the discretion of the investigator. The first cycle of chemotherapy was started on day 1 of study enrolment; subsequent cycles were administered approximately every 4 weeks. The maximum study duration was 26 weeks. All patients were required to be ambulatory and at least capable of self care – World Health Organization (WHO) performance score ≤ 2 (Miller et al, 1981). Further inclusion criteria were: Hb level ≥ 10.5 g $dl^{-1}$, neutrophil count > 3000 $mm^{-3}$, platelet count > 100 000 $mm^{-3}$, no clinically relevant abnormalities of renal or hepatic function, serum calcium < 10.6 mg $dl^{-1}$ and stool samples negative for occult blood. Patients were excluded if they were pregnant or of childbearing potential and not taking adequate contraceptive measures, or if they had any clinically significant disease, a history of primary haematological disease, anaemia attributable to factors other than cancer and/or chemotherapy, cerebral metastases, uncontrolled hypertension, a history of seizures or acute illness within 7 days of study entry. In addition, patients who had received androgen therapy within 2 months of study entry and those who had received any experimental treatment, immunosuppressive drugs, or other agents known to affect haematocrit, within 1 month prior to study entry, were excluded, as were patients receiving haematopoietic growth factors (including epoetin α) and those participating in another clinical trial.

The study was performed in accordance with the Declaration of Helsinki (1964) revised in Tokyo (1975), and the subsequent Venice (1983) and Hong Kong (1989) amendments. Approval of the protocol by local Ethical Review Committees was also obtained.

After providing written informed consent, patients were randomized to one of three groups: subcutaneous (s.c.) epoetin α 150 or 300 IU $kg^{-1}$ three times per week (at intervals of 2–3 days), or no epoetin α (untreated control). It was considered inappropriate to give placebo injections to the control group because of the increased risk of thrombocytopenia-related bruising. Epoetin α (Eprex™, Procrit®, Erypo®) was supplied by the RW Johnson Pharmaceutical Research Institute. Treatment with epoetin α was started 1 day after administration of each cycle of chemotherapy and continued until 3 days prior to the following cycle; treatment continued for 1 month after the final cycle. If the Hb level exceeded 15 g $dl^{-1}$, epoetin α was discontinued until the value had fallen to < 13 g $dl^{-1}$, at which point treatment was reinstated at half the initial dose. Transfusions were allowed as necessary, e.g. for symptomatic anaemia. Concomitant use of androgens was not permitted, and no patient received iron supplementation during the study.

Baseline assessments included medical history and physical examination, a listing of all current therapies, determination of vital signs, a 12-lead electrocardiogram (ECG) recording and laboratory parameters, a QOL questionnaire in which patients' responses to three questions (energy level, daily activity and overall QOL) were scored on a 100 mm visual analogue scale, and WHO performance score. Bone marrow aspirates and trephine biopsies were performed only if they formed part of the normal staging procedure prior to chemotherapy. Serum EPO levels were determined at the start of the first chemotherapy cycle only. Laboratory parameters and concomitant therapies were documented at the start of each cycle; haematological parameters were also assessed mid-cycle and, if the following cycle was delayed, at the planned start of the next cycle. Vital signs and adverse events were also determined after each s.c. injection of epoetin α. Final evaluations at study end included a physical examination, information regarding study withdrawal, QOL indices and WHO performance score, the physician's global assessment, a 12-lead ECG recording and assessment of laboratory parameters. The presence of epoetin α antibodies was determined both at the start of the study and at completion.

### Assessment of efficacy

The primary efficacy assessment was the prevention of anaemia, defined as the maintenance of a Hb level ≥ 10 g $dl^{-1}$. Secondary parameters were the number of RBC units transfused per patient and per cycle, and the pattern of Hb and haematocrit levels during the course of chemotherapy. Patient well-being in the week prior to each cycle of chemotherapy was assessed by the QOL questionnaire and WHO performance score. Safety assessments included patient discontinuation information, vital signs (recorded in the treated groups only) and the incidence and severity of adverse events, laboratory parameters at the start of each cycle, and epoetin α antibody titre at study end compared to baseline.

### Statistical analyses

Comparability of the three groups with regard to demographic and clinical characteristics at baseline was tested by means of analysis

*© Cancer Research Campaign 1999*

of variance (ANOVA), Kruskal–Wallis or $\chi^2$ tests, as appropriate. Differences between treatment groups for mid-cycle Hb and haematocrit through cycles 1–6 were tested using ANOVA. Within-group differences from baseline for efficacy parameters were tested using a paired Student's *t*-test. The proportion of patients transfused was compared between treatment groups using a Cochran–Mantel–Haenszel analysis. For pairwise comparison of treatment groups, the sequentially rejective Bonferroni–Holm procedure was applied to adjust for the three multiple comparisons. The time to become anaemic or require transfusion was analysed by survival analysis using Kaplan–Meier estimates and the log-rank test. All tests were conducted at the two-sided, 0.05 significance level.

### RESULTS

A total of 130 patients were enrolled (untreated, $n$ = 44; epoetin α 150 IU kg$^{-1}$, $n$ = 42; epoetin α 300 IU kg$^{-1}$, $n$ = 44). All patients were eligible for inclusion in the efficacy analysis.

The demographic and clinical characteristics of the three groups at baseline are summarized in Table 1. Overall, there were no statistically significant between-group differences. Hb levels were within the normal range and neutrophil and reticulocyte counts were not depressed. The majority of patients received cytotoxic drug regimens containing either carboplatin (82%) or cisplatin (7%). The most frequently administered combination ($n$ = 39) was the VICE regimen (etoposide 120 mg m$^{-2}$ intravenously (i.v.) on days 1 and 2 (followed by 240 mg m$^{-2}$ orally on day 3); carboplatin 300 mg m$^{-2}$ i.v. and ifosfamide 5 g m$^{-2}$ i.v. on day 1; and vincristine 1 mg on day 14). The majority of patients ($n$ = 69) received combinations of two or three of these agents or cisplatin plus etoposide. Other chemotherapeutic agents used during the study included doxorubicin, cyclophosphamide, epirubicin, methotrexate and lomustine.

More than 75% of patients completed at least 4 cycles of chemotherapy, with a mean number of 4.7 cycles per patient, 80% of cycles were administered on time. Cycle length was comparable between treatment groups and the number of patients per cycle in each group declined at approximately the same rate, suggesting that a similar drop out in these patient groups would be unlikely to influence the results. All patients were, therefore, evaluated for efficacy according to cycle.

In total, 42 patients prematurely discontinued the study (Table 2). The most common reasons for study withdrawal were death (nine patients), adverse events (seven patients) and other reasons (22 patients). The number of patients withdrawn was comparable in the three groups.

Epoetin α dosage reductions, required at some time during the study because of an increase in Hb level to >15 g dl$^{-1}$, was required in nine patients (21%) initially treated with epoetin α 150 IU kg$^{-1}$ and in 17 (39%) of the epoetin α 300 IU kg$^{-1}$ group. As a result of the omission of treatment with epoetin α during chemotherapy in some patients, the mean weekly dosages of epoetin α in the two groups were 335 and 612 IU kg$^{-1}$ respectively.

### Transfusion requirements and haematological response

A significantly higher percentage of patients in the untreated group experienced anaemia (Hb < 10 g dl$^{-1}$) during the study (66%) compared with patients treated with either epoetin α 150 IU kg$^{-1}$ (48%; $P$ < 0.05) or 300 IU kg$^{-1}$ (39%; $P$ = 0.005). The difference was particularly apparent during cycles 2–5, but then became less evident (Figure 1).

The probability of patients requiring no transfusions during the study is shown in Figure 2. The beneficial effect of epoetin α was evident by the second cycle of treatment and persisted throughout the study. Overall, 26/44 patients (59%) in the untreated group required

**Table 1** Demographic and clinical characteristics at baseline

| Parameter | Untreated ($n$ = 44) | Epoetin α 150 IU kg$^{-1}$ ($n$ = 42) | Epoetin α 300 IU kg$^{-1}$ ($n$ = 44) |
|---|---|---|---|
| Male/female ($n$) | 27/17 | 26/16 | 29/15 |
| Median age, years (range) | 60.0 (39–74) | 59.0 (43–72) | 58.5 (30–72) |
| Median weight, kg (range) | 63.8 (44–108) | 65.2 (43–107) | 70.8 (42–95) |
| Median height, cm (range) | 166.5 (150–185) | 168.0 (145–189) | 172.0 (150–187) |
| Median Hb level, g dl$^{-1}$ (range) | 13.4 (10.9–16.4) | 13.7 (10.7–16.1) | 13.6 (10.9–17.0) |
| Median Hct, % (range) | 39.4 (32.3–46.8) | 41.0 (32.6–50.3) | 40.0 (31.8–49.4) |
| Median reticulocyte count, × $10^9$ l$^{-1}$ (range) | 39.3 (0.1–109.1) | 40.1(1.0–76.2) | 42.0 (5.1–112.8) |
| Median neutrophil count, × $10^9$ l$^{-1}$ (range) | 5.9 (2.9–16.4) | 6.0 (1.7–11.3) | 6.3 (2.6–14.9) |
| Median WHO performance score, 0–4 (range) | 1.0 (0–2) | 1.0 (0–3) | 1.0 (0–2) |
| Median QOL scores, 0–100 mm (range) | | | |
| Energy level | 51.0 (0–94) | 47.0 (11–100) | 52.5 (0–100) |
| Daily activity | 32.0 (0–97) | 46.0 (5–100) | 49.0 (0–100) |
| Overall QOL | 49.0 (0–98) | 44.0 (1–100) | 50.5 (0–100) |
| Chemotherapy regimen ($n$) | | | |
| Carboplatin-based | 38 | 34 | 35 |
| Cisplatin-based | 2 | 2 | 5 |
| Other | 4 | 6 | 4 |

Hb = haemoglobin; Hct = haematocrit; QOL = quality of life; WHO = World Health Organization.

© Cancer Research Campaign 1999

**Table 2** Reasons for premature study discontinuation

| Parameter | Untreated ($n$ = 44) | Epoetin α 150 IU kg$^{-1}$ ($n$ = 42) | Epoetin α 300 IU kg$^{-1}$ ($n$ = 44) |
|---|---|---|---|
| Adverse events | 2 | 4 | 1 |
| Death | 3 | 1 | 5 |
| Intercurrent illness | 1 | 1 | 2 |
| Other[a] | 8 | 10 | 4 |
| Total | 14 (32%) | 16 (38%) | 12 (27%) |

[a]Including personal reasons, loss to follow-up, non-responder to chemotherapy, disease progression or remission, discontinuation of chemotherapy, toxicity of chemotherapy, elevated haemoglobin, deterioration of general condition and physician decision.

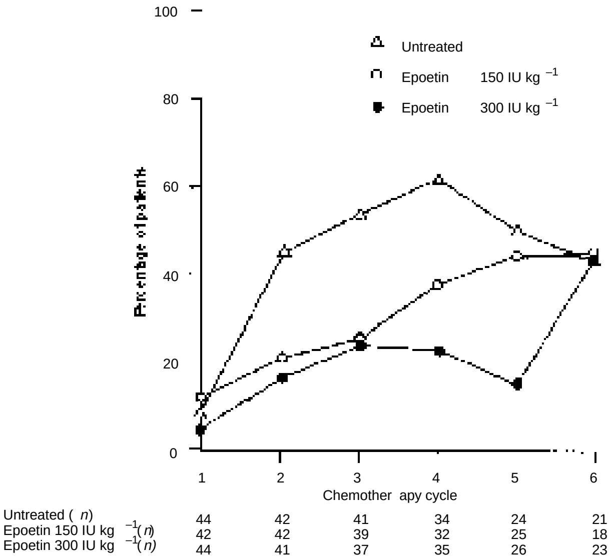

**Figure 1** Effect of epoetin α on the percentage of patients becoming anaemic (haemoglobin level < 10 g dl$^{-1}$) during cyclic combination (primarily platinum-based) chemotherapy for small cell lung cancer; $n$ = number of patients

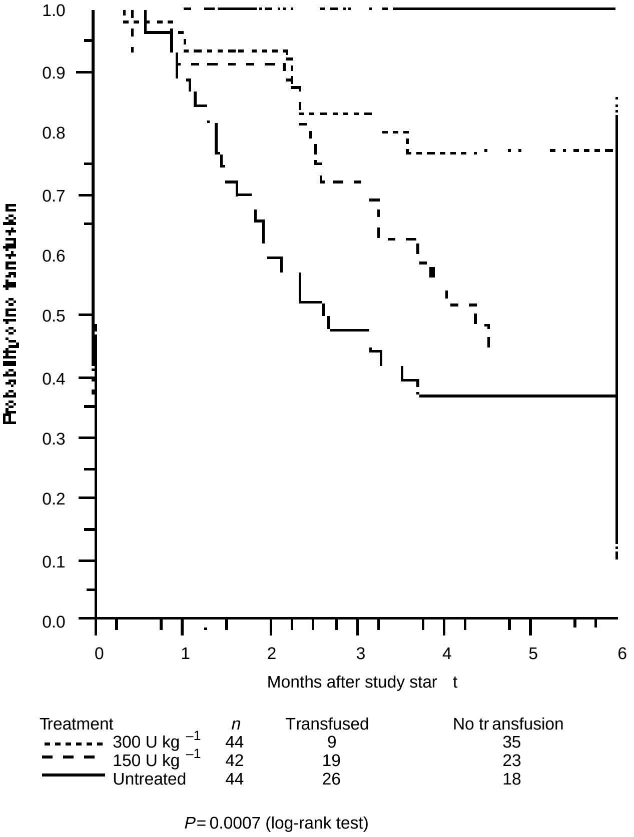

**Figure 2** Statistically significant effect ($P$ = 0.0007; log-rank test) of epoetin α on the probability of patients not requiring transfusions during cyclic combination (primarily platinum-based) chemotherapy for small cell lung cancer transfusions compared with 19/42 patients (45%) in the epoetin α 150 IU kg$^{-1}$ group ($P < 0.05$) and only 9/44 (20%) in the epoetin α 300 IU kg$^{-1}$ group ($P < 0.001$). The difference between the two epoetin α groups was significant ($P < 0.005$). The total number of transfusions administered in the three groups was 73, 41 and 25 respectively. The mean cumulative transfusion rate for the six cycles was also significantly higher in the untreated group than in either the epoetin α 150 IU kg$^{-1}$ or 300 IU kg$^{-1}$ groups ($P < 0.01$ and $P < 0.001$ respectively) (Table 3). The difference between the two epoetin α groups was also significant ($P < 0.05$). The mean Hb level at first transfusion varied between centres, ranging from 8.4 to 12.9 g dl$^{-1}$ for the 150 IU kg$^{-1}$ group, 8.7 to 10.8 g dl$^{-1}$ for the 300 IU kg$^{-1}$ group, but 8.2 to 10.0 g dl$^{-1}$ for the untreated group.

Mean Hb levels by cycle are shown in Figure 3. During the first cycle, mean Hb did not differ significantly from baseline in any group. However, during the second, third and fourth cycles there were significant between-group differences for the reduction in Hb level from baseline. Epoetin α not only reduced the mid-cycle Hb nadir, but also significantly delayed the onset of anaemia (Hb < 10 g dl$^{-1}$) and/or the first RBC transfusion ($P < 0.01$). The median time to become anaemic/require transfusion was 59/48, 116/98 and > 142/166 days in the untreated, epoetin α 150 IU kg$^{-1}$ and 300 IU kg$^{-1}$ groups respectively. Mean haematocrit values and changes from baseline followed a similar pattern to that of Hb levels (data not shown).

In order to assess the possibility that variations in the intensity of chemotherapy accounted for the between-group differences in Hb values, transfusion rates and the number of patients becoming anaemic or becoming anaemic and/or requiring transfusion, the proportion of patients with neutrophil counts $< 1 \times 10^9$ l$^{-1}$ was determined as a surrogate marker of chemotherapy intensity. There were no statistically significant differences between the treatment groups with respect to the incidence of marked neutropenia (Table 4), suggesting that there were no differences in chemotherapy intensity. In addition, the potential effects of differential transfusion practices were determined from the mean Hb value within 7 days prior to transfusion. The results showed that patients in the epoetin α 300 IU kg$^{-1}$ groups were transfused at lower Hb levels (8.0 g dl$^{-1}$) than in either the epoetin α 150 IU kg$^{-1}$ group (8.6 g dl$^{-1}$) or untreated patients (8.5 g dl$^{-1}$), suggesting that patients in the 300 IU kg$^{-1}$ group may have been somewhat under-transfused compared with patients in other groups.

### QOL and WHO performance scores

Parameters assessed by the QOL questionnaire, namely energy level, daily activity and overall QOL, did not show any marked

*© Cancer Research Campaign 1999*

**Table 3** Mean (± s.d.) cumulative transfusion rate for the six cycles and per patient, by treatment group

| Cumulative transfusion rate | Untreated ($n$ = 44) | Epoetin α 150 IU kg$^{-1}$ ($n$ = 42) | Epoetin α 300 IU kg$^{-1}$ ($n$ = 44) |
|---|---|---|---|
| Units per six cycles | 6.13 ± 7.13 | 3.84 ± 5.58 | 2.10 ± 4.60 |
| Units per patient | | | |
| 0 | 18 | 23 | 35 |
| 1–5 | 6 | 7 | 1 |
| > 5–10 | 7 | 6 | 4 |
| > 10–15 | 9 | 4 | 3 |
| > 15 | 4 | 2 | 1 |

**Table 4** Number of patients with neutrophil counts <1 × 10$^{9}$l$^{-1}$ by cycle and treatment group

| Cycle | Untreated | Epoetin α 150 IU kg$^{-1}$ | Epoetin α 300 IU kg$^{-1}$ |
|---|---|---|---|
| 1 | 19/44 (43%) | 21/42 (50%) | 22/44 (50%) |
| 2 | 22/42 (52%) | 18/42 (43%) | 25/41 (61%) |
| 3 | 16/41 (39%) | 14/39 (36%) | 22/37 (59%) |
| 4 | 14/34 (41%) | 13/32 (41%) | 17/35 (49%) |
| 5 | 8/24 (33%) | 12/25 (48%) | 16/26 (62%) |
| 6 | 8/21 (38%) | 8/18 (44%) | 16/23 (70%) |
| Total | 30/44 (68%) | 27/42 (64%) | 37/44 (84%) |

changes from baseline at the end of the study in any group, with the exception of a significant improvement in overall QOL in the epoetin α 150 IU kg$^{-1}$ group ($P$ < 0.05). There were no significant between-group differences (Table 5), which may have been related to the fact that all groups had similar Hb values at study end (approximately 10–11 g dl$^{-1}$). In this study of lung cancer patients it was considered reasonable not to insist on a dogmatic haemoglobin level for transfusion and deny the patient the benefits of transfusion when needed, particularly in the control group. Not surprisingly, given this policy, QOL at the end of the study was similar in the three groups. Evaluation of WHO performance scores revealed similar findings, with no significant between- or within-group differences.

### Tolerability

Both dosages of epoetin α were well-tolerated. As shown in Table 6, the incidence and distribution of adverse events did not differ markedly between the three groups. Moreover, the only adverse event considered definitely related to epoetin α was a localized swelling and burning sensation at the injection site in two patients treated with the higher dosage. Premature discontinuation as a result of adverse events was not influenced by epoetin α treatment (Table 2). Myocardial infarction was documented in two patients receiving epoetin α 300 IU kg$^{-1}$, but the relationship to therapy was unclear. None of the six deaths among epoetin α-treated patients were causally related to treatment.

There was no evidence of a sustained increase in blood pressure with either dosage of epoetin α. Among patients treated with epoetin α 150 IU kg$^{-1}$, one patient had several recordings of diastolic blood pressure around 105 mmHg, while another patient with a history of hypertension experienced an elevation of blood pressure to 180/120 mmHg after the second dose. A third patient, treated with epoetin α 300 IU kg$^{-1}$, developed moderate hypertension (up to 180/115 mmHg) that regressed after the institution of

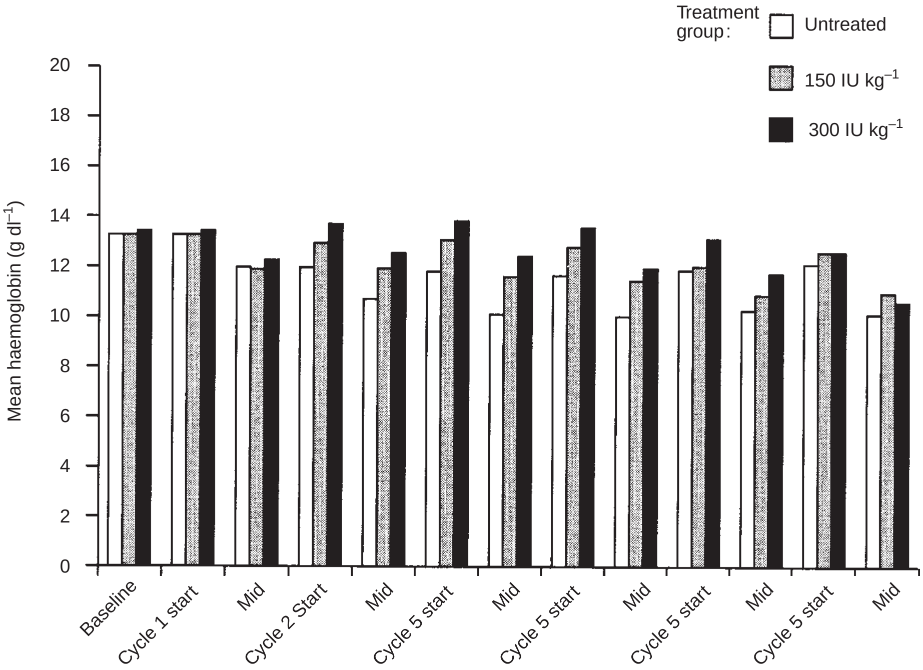

**Figure 3** Effect of epoetin α on mean haemoglobin (Hb) levels at baseline, start of cycle and mid-cycle, in 130 patients receiving cyclic combination (primarily platinum-based) chemotherapy for small cell lung cancer

 © Cancer Research Campaign 1999

**Table 5** Mean (± s.d.) baseline and change to end-point measurements of QOL parameters[a]

| QOL parameter (0–100 mm) | Untreated | | Epoetin α 150 IU kg$^{-1}$ | | Epoetin α 300 IU kg$^{-1}$ | |
|---|---|---|---|---|---|---|
| | Baseline (*n* = 37) | Change (*n* = 27) | Baseline *n* = 37) | Change (*n* = 33) | Baseline (*n* = 38) | Change (*n* = 32) |
| Energy level | 48.4 ± 23.6 | 1.6 ± 23.9 | 53.6 ± 27.7 | –2.3 ± 31.9 | 50.7 ± 28.4 | 3.2 ± 40.1 |
| Daily activity | 41.7 ± 28.1 | 10.8 ± 35.6 | 50.8 ± 29.3 | 3.0 ± 31.7 | 50.8 ± 29.5 | 4.9 ± 38.5 |
| Overall QOL | 47.9 ± 26.7 | 7.5 ± 29.1 | 49.0 ± 28.1 | 11.7 ± 30.6[b] | 53.9 ± 28.3 | 6.0 ± 41.0 |

[a]Positive values indicate an improvement. QOL = quality of life; [b] $P < 0.05$ vs baseline.

**Table 6** Adverse events reported by ≥ 5% of patients in any treatment group

| Adverse event | Untreated (*n* = 44) | Epoetin α 150 IU kg$^{-1}$ (*n* = 42) | Epoetin α 300 IU kg$^{-1}$ (*n* = 44) |
|---|---|---|---|
| Anaemia | 19 (43%) | 14 (33%) | 10 (23%) |
| Thrombocytopenia | 9 (20%) | 11 (26%) | 9 (20%) |
| Bacterial infection | 10 (23%) | 8 (19%) | 7 (16%) |
| Nausea | 6 (14%) | 3 (7%) | 7 (16%) |
| Neutropenia | 8 (18%) | 5 (12%) | 6 (14%) |
| Pyrexia | 7 (16%) | 7 (17%) | 5 (11%) |
| Dyspnoea | 1 (2%) | 1 (2%) | 5 (11%) |
| Vomiting | 5 (11%) | 5 (12%) | 4 (9%) |
| Dizziness | 1 (2%) | 3 (7%) | 4 (9%) |
| Cough | 0 | 0 | 4 (9%) |
| Headache | 1 (2%) | 2 (5%) | 3 (7%) |
| Constipation | 1 (2%) | 2 (5%) | 3 (7%) |
| Malaise | 0 | 2 (5%) | 3 (7%) |
| Urinary tract infection | 0 | 0 | 3 (7%) |
| Alopecia | 3 (7%) | 1 (2%) | 2 (5%) |
| Oedema | 0 | 4 (10%) | 1 (2%) |
| Diarrhoea | 2 (5%) | 5 (12%) | 1 (2%) |
| Rash | 4 (9%) | 5 (12%) | 1 (2%) |
| Decreased WBC count | 3 (7%) | 1 (2%) | 1 (2%) |
| Lethargy | 3 (7%) | 1 (2%) | 0 |

anti-hypertensive therapy; this event may have been drug-related. Overall, there was a significant reduction in mean systolic blood pressure over time in both epoetin α treatment groups ($P \leq 0.01$).

Low serum iron and transferrin saturation values were seen in several patients, mainly in the low-dosage epoetin α group. In some cases this may have been caused by ACD combined with iron deficiency, as mean ferritin levels were normal or elevated. There were no clinically significant, treatment-related changes in physical examination, serum chemistry parameters or the ECG. In addition, no antibodies against epoetin α developed during treatment.

### DISCUSSION

SCLC progresses rapidly in the absence of therapy. The optimal response rates are achieved with combination chemotherapy programmes. However, such treatment regimens are associated with a significant level of myelosuppression that may necessitate frequent blood transfusions, with their attendant problems.

The results of this open-label, controlled study demonstrate the beneficial effects of treatment with epoetin α in patients undergoing cyclic, primarily platinum-based, combination chemotherapy for SCLC. Indeed, patients receiving epoetin α required significantly fewer transfusions and had a significantly lower mean cumulative transfusion rate than untreated patients. These findings were corroborated by assessment of the haematological response. Although patients had Hb levels that were either normal or bordering on anaemia at the start of the study, the myelosuppressive effects of chemotherapy were already evident after the first cycle of treatment. By the second cycle, however, the protective effects of epoetin α became apparent, with significantly higher Hb levels in both epoetin α-treated groups than in untreated patients. This time-course of events was as expected, in view of the fact that an increase in reticulocytes is usually seen 3–4 days after initiation of epoetin α-stimulated erythropoiesis, with the Hb response following more slowly thereafter. The maintenance of a higher Hb level in the epoetin α-treated groups continued through the third and fourth cycles of treatment, after which the differences between the groups became somewhat less marked, possibly as a result of heavier use of transfusions in the control group. Alternatively, since iron supplementation was not administered during this study, iron deficiency in later cycles may have led to a reduction in epoetin α efficacy (Eschbach et al, 1978). Moreover, drop-outs as chemotherapy cycles progressed, or problems resulting from repeated chemotherapy, such as neutropenic sepsis, may have blunted the effects of epoetin α. The possibility that variations in the intensity of chemotherapy may have explained the differences in Hb values and transfusion rates between groups was evaluated and considered not significant. The threshold for transfusion clearly depends on symptoms in a vulnerable patient group such as lung cancer. The tolerability of dyspnoea and lethargy together with local transfusion policies which differ from centre to centre and from country to country mitigated against a rigid transfusion policy, e.g. haemoglobin of 8 g dl$^{-1}$ or less. However, we did obtain data for differences between centres who regard transfusion trigger policies. A somewhat lower mean Hb level prior to transfusion in the epoetin α 300 IU kg$^{-1}$ group, may have meant that patients in this treatment group were actually under-transfused for the degree of anaemia. Thus, the efficacy of the epoetin α 300 IU kg$^{-1}$ regimen may have been exaggerated compared with the 150 IU kg$^{-1}$ regimen.

The absence of marked improvements in measures of QOL and WHO performance score at study end compared with baseline was not unexpected in view of the similar mean Hb levels in the three groups during the last cycle. Also, these measurements were most likely influenced by the design of the study as epoetin α therapy was initiated before, rather than after, the onset of anaemia. Again, any differences between groups may have been masked by the administration of blood transfusions or the effects of repeated chemotherapy.

*© Cancer Research Campaign 1999* 

In common with previously published findings (Ludwig et al, 1990; Abels, 1993), treatment with both dosages of epoetin α showed excellent tolerability throughout the study. The incidence and type of adverse events did not differ markedly between the three groups, and epoetin α had no effect on the rate of premature study discontinuation. A local swelling and burning sensation at the site of injection was the only adverse event considered definitely related to epoetin α treatment. This effect was seen in just two patients, both of whom were in the 300 IU $kg^{-1}$ group, and may have been associated with large injection volumes or insufficient warming of the solution to room temperature before use.

In contrast to anaemia of end-stage renal disease, in which 20–30% of patients treated with epoetin α experience hypertensive episodes (Radermacher and Koch, 1993), there was no evidence of a significant increase in blood pressure in this study. In fact, there was an overall significant reduction in mean systolic blood pressure over time in both epoetin α treatment groups. There was no indication of any other cardiovascular complications with epoetin α therapy. However, significant hyptertension arising de novo, and responsive to appropriate treatment, may have been related to epoetin α therapy in one patient. While such findings suggest that hypertension can occur in cancer patients treated with epoetin α, this is much less common than in patients treated with epoetin α for renal anaemia.

The low serum iron and transferrin saturation seen in some patients was thought to be related to ACD, combined with iron deficiency. As epoetin α-accelerated erythropoiesis utilizes a considerable amount of iron (Eschbach et al, 1978), patients may require iron supplementation; the management of such supplementation may well have been inadequate in this study and should be considered in subsequent investigations.

As response and survival among cancer patients may depend on the administration of dose-intensive combination chemotherapy, it is important that effective treatments are available to alleviate (or even prevent) the toxic effects of such intensive treatment. This study has demonstrated that epoetin α is effective and well-tolerated in maintaining Hb level ≥ 10 g $dl^{-1}$ and reducing transfusion requirements in patients with SCLC undergoing primarily platinum-based, cyclic combination chemotherapy. Consequently, exposure to the risks associated with transfusion, such as infection transmission, allergic reactions, iron overload and immunosupression, is reduced and the patient may be able to tolerate higher levels of chemotherapy. These significant clinical benefits were achieved with mean weekly doses of epoetin α 335 and 612 IU $kg^{-1}$ in the two treatment groups. Consequently, a dosage regimen of 150 IU $kg^{-1}$ s.c. three times a week is appropriate, with escalation to 300 IU $kg^{-1}$, if necessary.

### REFERENCES

Abels R (1993) Erythropoietin for anaemia in cancer patients. *Eur J Cancer* **29A**: S2–8

Baer AN, Dessypris E, Goldwasser E and Krantz SB (1987) Blunted erythropoietin response to anaemia in rheumatoid arthritis. *Br J Haematol* **66**: 559–564

Blumberg N and Heal JM (1994) Effects of transfusion on immune function. Cancer recurrence and infection. *Arch Pathol Lab Med* **118**: 371–379

Canetta R, Rosencweig M and Carter SK (1985) Carboplatin: the clinical spectrum to date. *Cancer Treat Rev* **12**: 125–136

Cascinu S, Fedeli A, Fedeli SL and Catalano G (1993) Cisplatin-associated anaemia treated with subcutaneous erythropoietin. A pilot study. *Br J Cancer* **67**: 156–158

Case DC, Bukowski RM, Carey RW, Fishkin EH, Henry DH, Jacobson RJ, Jones SE, Keuer AM, Kugler JW, Nichols CR, Salmon SE, Silver RT, Storniolo AM, Wampler GL, Dooley CM, Larholt KM, Nelson RA and Abels RI (1993) Recombinant human erythropoietin therapy for anemic cancer patients on combination chemotherapy. *J Natl Cancer Inst* **85**: 801–806

de Campos E, Radford J, Steward W, Milroy R, Dougal M, Swinden R, Testa N and Thatcher N (1995) Clinical and in vitro effects of recombinant human erythropoietin in patients receiving intensive chemotherapy for small cell lung cancer. *J Clin Oncol* **13**: 1623–1631

Del Mastro L, Venturini M, Lionetto R, Garrone O, Melioli G, Pasquetti W, Sertoli MR, Bertelli G, Canavese G, Constantini M and Rosso R (1997) Randomized phase III trial evaluating the role of erythropoietin in the prevention of chemotherapy-induced anemia. *J Clin Oncol* **15**: 2715–2721

Eschbach JW, Egrie JC, Downing MR, Browne JK and Adamson JW (1978) Correction of the anemia of end-stage renal disease with recombinant human erythropoietin. *N Engl J Med* **316**: 73–78

Gebbia V, Valenza R and Rausa L (1990) The in vitro effect of recombinant erythropoietin on cisdiamminodichloroplatinum-induced inhibition of murine erythroid stem cells. *Anticancer Res* **10**: 1779–1782

Glaspy J, Bukowski R, Steinberg D, Taylor C, Tchekmedyian S and Vadhan-Raj S (1997) Impact of therapy with epoetin alfa on clinical outcomes in patients with nonmyeloid malignancies during cancer chemotherapy in community oncology practice Procrit Study Group. *J Clin Oncol* **15**: 1218–1234

Henry DH, Rudnick SA, Bryant E, Adels RI, Danna RP, Staddon AP and Mason BA (1989) Preliminary report of two double-blind, placebo-controlled studies using human recombinant erythropoietin (r-HuEPO) in the anemia associated with cancer. *Blood* **74**: 6a

Hesketh PJ, Cooley TP, Finkel HE, Wright J and Hesketh AM (1988) Treatment of advanced non-small cell lung cancer with cisplatin, 5-fluorouracil and mitomycin C. *Cancer* **62**: 1466–1470

Kaye SB, Lewis CR, Paul J, Duncan ID, Gordon HK, Kitchener HC, Cruickshank DJ, Atkinson RJ, Soukop M, Rankin EM, Cassidy J, Davis JA, Reed NS, Crawford SM, MacLean A, Swapp GA, Sarkar TK, Kennedy JH and Symonds RP (1992) Randomised study of two doses of cisplatin with cyclophosphamide in epithelial ovarian cancer. *Lancet* **340**: 329–333

Kuzur ME and Greco FA (1980) Cisplatin-induced anemia. *N Engl J Med* **303**: 110–111

Ludwig H, Fritz E, Kotzmann H, Hocker P, Gisslinger H and Barnas U (1990) Erythropoietin treatment of anemia associated with multiple myeloma. *N Engl J Med* **322**: 1693–1699

Ludwig H, Leitgeb C, Fritz E, Krainer M, Kuhrer I, Kornek G, Sagaster P and Weissmann A (1993*a*) Erythropoietin treatment of chronic anaemia of cancer. *Eur J Cancer* **29A**: S8–12

Ludwig H, Pecherstorfer M, Leitgeb C and Fritz E (1993*b*). Recombinant human erythropoietin for the treatment of chronic anemia in multiple myeloma and squamous cell carcinoma. *Stem Cells* **11**: 348–355

Miller AB, Hoogstraten B, Staquet M and Winkler A (1981) Reporting results of cancer treatment. *Cancer* **47**: 207–214

Miller CB, Jones RJ, Piantadosi S, Abeloff MD and Spivak JL(1990) Decreased erythropoietin response in patients with the anemia of cancer. *N Engl J Med* **322**: 1689–1692

Radermacher J and Koch KM (1993) Erythropoietin and hypertension. In *Erythropoietin*: *Molecular Physiology and Clinical Applications*, Bauer C, Koch KM, Scigella P, et al (eds), pp. 129–152. Marcel Dekker: New York

Rothmann SA, Paul P, Weick JK, McIntyre WR and Fantelli F (1985) Effect of *cis*-diamminedichloroplatinum on erythropoietin production and hematopoietic progenitor cells. *Int J Cell Cloning* **3**: 415–423

Skillings JR, Sridhar FG, Wong C and Paddock L (1993) The frequency of red cell transfusion for anemia in patients receiving chemotherapy. A retrospective cohort study. *Am J Clin Oncol* **16**: 22–25

Walker RH (1987) Special report: transfusion risks. *Am J Clin Pathol* **88**: 374–378

© Cancer Research Campaign 1999

## References

[PDF_00658] Abels R. (1993). Erythropoietin for anaemia in cancer patients.. Eur J Cancer.

[PDF_00660] Baer A. N., Dessypris E. N., Goldwasser E., Krantz S. B. (1987). Blunted erythropoietin response to anaemia in rheumatoid arthritis.. Br J Haematol.

[PDF_00662] Blumberg N., Heal J. M. (1994). Effects of transfusion on immune function. Cancer recurrence and infection.. Arch Pathol Lab Med.

[PDF_00664] Canetta R., Rozencweig M., Carter S. K. (1985). Carboplatin: the clinical spectrum to date.. Cancer Treat Rev.

[PDF_00666] Cascinu S., Fedeli A., Fedeli S. L., Catalano G. (1993). Cisplatin-associated anaemia treated with subcutaneous erythropoietin. A pilot study.. Br J Cancer.

[PDF_00669] Case D. C., Bukowski R. M., Carey R. W., Fishkin E. H., Henry D. H., Jacobson R. J., Jones S. E., Keller A. M., Kugler J. W., Nichols C. R. (1993). Recombinant human erythropoietin therapy for anemic cancer patients on combination chemotherapy.. J Natl Cancer Inst.

[PDF_00678] Del Mastro L., Venturini M., Lionetto R., Garrone O., Melioli G., Pasquetti W., Sertoli M. R., Bertelli G., Canavese G., Costantini M. (1997). Randomized phase III trial evaluating the role of erythropoietin in the prevention of chemotherapy-induced anemia.. J Clin Oncol.

[PDF_00682] Eschbach J. W., Egrie J. C., Downing M. R., Browne J. K., Adamson J. W. (1987). Correction of the anemia of end-stage renal disease with recombinant human erythropoietin. Results of a combined phase I and II clinical trial.. N Engl J Med.

[PDF_00685] Gebbia V., Valenza R., Rausa L. (1990). The in vitro effect of recombinant erythropoietin on cisdiamminodichloroplatinum-induced inhibition of murine erythroid stem cells.. Anticancer Res.

[PDF_00688] Glaspy J., Bukowski R., Steinberg D., Taylor C., Tchekmedyian S., Vadhan-Raj S. (1997). Impact of therapy with epoetin alfa on clinical outcomes in patients with nonmyeloid malignancies during cancer chemotherapy in community oncology practice. Procrit Study Group.. J Clin Oncol.

[PDF_00696] Hesketh P. J., Cooley T. P., Finkel H. E., Wright J., Hesketh A. M. (1988). Treatment of advanced non-small cell lung cancer with cisplatin, 5-fluorouracil, and mitomycin C.. Cancer.

[PDF_00699] Kaye S. B., Lewis C. R., Paul J., Duncan I. D., Gordon H. K., Kitchener H. C., Cruickshank D. J., Atkinson R. J., Soukop M., Rankin E. M. (1992). Randomised study of two doses of cisplatin with cyclophosphamide in epithelial ovarian cancer.. Lancet.

[PDF_00704] Kuzur M. E., Greco F. A. (1980). Cisplatin-induced anemia.. N Engl J Med.

[PDF_00706] Ludwig H., Fritz E., Kotzmann H., Höcker P., Gisslinger H., Barnas U. (1990). Erythropoietin treatment of anemia associated with multiple myeloma.. N Engl J Med.

[PDF_00709] Ludwig H., Leitgeb C., Fritz E., Krainer M., Kührer I., Kornek G., Sagaster P., Weissmann A. (1993). Erythropoietin treatment of chronic anaemia of cancer.. Eur J Cancer.

[PDF_00712] Ludwig H., Pecherstorfer M., Leitgeb C., Fritz E. (1993). Recombinant human erythropoietin for the treatment of chronic anemia in multiple myeloma and squamous cell carcinoma.. Stem Cells.

[PDF_00715] Miller A. B., Hoogstraten B., Staquet M., Winkler A. (1981). Reporting results of cancer treatment.. Cancer.

[PDF_00717] Miller C. B., Jones R. J., Piantadosi S., Abeloff M. D., Spivak J. L. (1990). Decreased erythropoietin response in patients with the anemia of cancer.. N Engl J Med.

[PDF_00723] Rothmann S. A., Paul P., Weick J. K., McIntyre W. R., Fantelli F. (1985). Effect of cis-diamminedichloroplatinum on erythropoietin production and hematopoietic progenitor cells.. Int J Cell Cloning.

[PDF_00726] Skillings J. R., Sridhar F. G., Wong C., Paddock L. (1993). The frequency of red cell transfusion for anemia in patients receiving chemotherapy. A retrospective cohort study.. Am J Clin Oncol.

[PDF_00729] Walker R. H. (1987). Special report: transfusion risks.. Am J Clin Pathol.

[PDF_00674] de Campos E., Radford J., Steward W., Milroy R., Dougal M., Swindell R., Testa N., Thatcher N. (1995). Clinical and in vitro effects of recombinant human erythropoietin in patients receiving intensive chemotherapy for small-cell lung cancer.. J Clin Oncol.

